# Hurricane Sandy: How Did We Do? Assessing a Manhattan Hospital’s Response

**DOI:** 10.3389/fpubh.2014.00090

**Published:** 2014-07-25

**Authors:** Christina Ngoc Tram Tran, Michael Heller, Abraham Berger, Joseph Habboushe

**Affiliations:** ^1^Emergency Department, Mount Sinai Beth Israel, New York, NY, USA

**Keywords:** Hurricane Sandy, Beth Israel Medical Center, Mount Sinai Beth Israel, hurricanes, disaster preparedness, emergency department, hurricane, visiting clinicians

Hurricane Sandy, the largest, most deadly, and second costliest Atlantic storm recorded in history, hit New York City the night of October 29, 2012 (1). The lights blacked out and a silence fell across the usually bustling streets of downtown Manhattan. Sandy had resulted in widespread flooding of the streets, major tunnels, and subway systems in lower Manhattan and left some 2.5 million residents in New Jersey and 2.3 million residents in New York without electricity (2). Flooding and generator failures led to the unprecedented evacuation and closure of all hospitals in the lower half of Manhattan except for Beth Israel Medical Center (3). As patients and ambulances arrived at the already busy emergency department (ED), Beth Israel was forced to face a new and unexpected challenge.

Beth Israel Medical Center is an urban teaching hospital with a 110K+ annual ED census with over 90 ED beds and a pre-hurricane daily inpatient census of 650 patients. As the only hospital left providing medical care in lower Manhattan, the ED census increased by 20% and ambulance arrivals spiked by 64% in the first 4 weeks. ED visits increased from a baseline of just under 300 daily to as many as 500 patients in the initial week of the disaster. The inpatient census increased to 750, with the hospital operating at full capacity. In an attempt to accommodate the increased volume, a third ED area was opened 24/7, a nearby rehab center was utilized as a venue for low-risk medical patients and beds were added to some two-person and three-person inpatient rooms, expanding total capacity by approximately 5%. However, in addition to total capacity increasing 5%, beds on the detox floors were converted to medicine beds, increasing medical admission capacity further. However, this was still not enough to accommodate the increase in volume, and lines formed out the door, resulting in a threefold increase (15%) of ambulance patients “left without being seen” (4). This larger-than-expected increase may be a cause of not only the increased waiting time but also the increased chaos and sense of frustration that was felt post-Hurricane. It may also reflect that a large portion of increased volume came from low-acuity patients, as one of the closest closed ED’s, at Bellevue Hospital, has a very low-acuity population with just an approximate 10% admission rate.

There was a large increase in the demand for social services as patients could not return to their flooded, waterless, and powerless homes, which was met by an immediate increased in social work presence. Social workers typically would see 8–10 patients/day, which spiked to approximately 200 in the first 3 days after the hurricane. Inpatient social work facilitated rapid discharge plans to make room for new patients. They helped to set up a building across the street as shelter and directed patients to other nearby shelters, and arranged transfers to assisted care facilities. This was helpful in reducing some of the crowding in the ED by mobilizing discharged patients.

The inpatient medicine floor was overwhelmed, resulting in long wait times for inpatient beds and boarding of patients in the ED for days. Medicine attending physicians visited admitted patients in the ED, and assisted with daily management. In a partially successful effort to alleviate ED overcrowding, representatives of renal, endocrine, and cardiology services also made “rounds” in the ED in an effort to avoid admissions for select patients who may safely be managed as outpatients. For example, some patients that would have been admitted for a cardiac work-up were immediately scheduled for next-day stress tests. There were also plans to admit non-surgical abdominal complaints to the surgical service although execution of these plans was not successful.

There was an early spike in the volume of oxygen-dependent patients, hemodialysis patients, and medication refills, with a total 33% increase from baseline. To streamline these HD patients, nephrologists came down to the ED and each patient immediately had a potassium level measured and quickly assessed for a need of emergent dialysis and admitted or transferred to a nearby facility. The Army Corps of Engineers brought generators to help the hospital and a nearby dialysis center was up and running within 3 days. With pharmacies closed, patients could not get prescriptions filled. The inpatient pharmacy began filling prescriptions for up to 5 days but quickly became overwhelmed and ran low on stock. Patients sometimes waited in the ED for hours to have their prescriptions filled.

Perhaps the most successful response was the increase in ED physician staffing by not only hospital staff but also by importing physicians from a nearby closed hospital. Native attending physicians provided an additional 197 h in the first week, an average 9 h additional per full time attending per week, with many working an additional 25 h/week. This resulted in two additional attendings during a weekday shift and three additional attendings during a weekend shift. With expedited credentialing, visiting physicians covered 63 h of these hours by week 3, somewhat reducing the burden. Similarly, native residents already in the ED worked extra shifts and those on off-service rotations were immediately pulled back to the ED, providing an additional 324 h of coverage, with each resident on average working an extra 12 h and residents pulled in from off-service rotations providing 48 h in the first week. Imported residents provided an additional 126 h/week by week 3. The imported providers stayed for 2 months, until mid-January. This was critical not only for Beth Israel hospital but also for the imported residents, who needed the clinical hours to meet national requirements to graduate (5). However, we found that visiting clinicians were less productive than home clinicians, by 10–30%, a known challenge of using visiting clinicians during emergencies (5).

Beth Israel has a 12-member Disaster Management Committee co-chaired by one physician and one non-physician leader. The former is the Vice Chair of the ED, and the latter carries a Masters in Public Health and had worked previously in EMS for the Fire Department of New York. The Disaster Management Committee also included hospital leadership, and would organize disaster management and run practice drills using standard command structure.

Hurricane Sandy was an extraordinary urban emergency challenge and Beth Israel was able to meet many unexpected obstacles applying creative and rapid solutions, reallocating resources, and providing a multifaceted response that was largely successful. However, there are still many opportunities for improvement, particularly in the area of pre-planning. With the invaluable lessons learned from this experience, Beth Israel Medical Center and New York City are already more prepared for their next disaster.

## Conflict of Interest Statement

The authors declare that the research was conducted in the absence of any commercial or financial relationships that could be construed as a potential conflict of interest.
